# Kinetic properties of “dual” orexin receptor antagonists at OX_1_R and OX_2_R orexin receptors

**DOI:** 10.3389/fnins.2013.00230

**Published:** 2013-12-03

**Authors:** Gabrielle E. Callander, Morenike Olorunda, Dominique Monna, Edi Schuepbach, Daniel Langenegger, Claudia Betschart, Samuel Hintermann, Dirk Behnke, Simona Cotesta, Markus Fendt, Grit Laue, Silvio Ofner, Emmanuelle Briard, Christine E. Gee, Laura H. Jacobson, Daniel Hoyer

**Affiliations:** ^1^Department of Pharmacology and Therapeutics, Faculty of Medicine, Dentistry and Health Sciences, School of Medicine, The University of MelbourneParkville, VIC, Australia; ^2^The Florey Institute of Neuroscience and Mental Health, The University of MelbourneParkville, VIC, Australia; ^3^Department of Neuroscience, Novartis Institutes for Biomedical ResearchBasel, Switzerland; ^4^Global Discovery Chemistry, Novartis Institutes for Biomedical ResearchBasel, Switzerland; ^5^Metabolism and Pharmacokinetics, Novartis Institutes for Biomedical ResearchBasel, Switzerland; ^6^Centre for Neurobiology Hamburg, Institute for Synaptic PhysiologyHamburg, Germany

**Keywords:** orexin receptor antagonists, dual orexin receptor antagonists, kinetics, radioligands

## Abstract

Orexin receptor antagonists represent attractive targets for the development of drugs for the treatment of insomnia. Both efficacy and safety are crucial in clinical settings and thorough investigations of pharmacokinetics and pharmacodynamics can predict contributing factors such as duration of action and undesirable effects. To this end, we studied the interactions between various “dual” orexin receptor antagonists and the orexin receptors, OX_1_R and OX_2_R, over time using saturation and competition radioligand binding with [^3^H]-BBAC ((S)-N-([1,1′-biphenyl]-2-yl)-1-(2-((1-methyl-1H-benzo[d]imidazol-2-yl)thio)acetyl)pyrrolidine-2-carboxamide). In addition, the kinetics of these compounds were investigated in cells expressing human, mouse and rat OX_1_R and OX_2_R using FLIPR® assays for calcium accumulation. We demonstrate that almorexant reaches equilibrium very slowly at OX_2_R, whereas SB-649868, suvorexant, and filorexant may take hours to reach steady state at both orexin receptors. By contrast, compounds such as BBAC or the selective OX_2_R antagonist IPSU ((2-((1*H*-Indol-3-yl)methyl)-9-(4-methoxypyrimidin-2-yl)-2,9-diazaspiro[5.5]undecan-1-one) bind rapidly and reach equilibrium very quickly in binding and/or functional assays. Overall, the “dual” antagonists tested here tend to be rather unselective under non-equilibrium conditions and reach equilibrium very slowly. Once equilibrium is reached, each ligand demonstrates a selectivity profile that is however, distinct from the non-equilibrium condition. The slow kinetics of the “dual” antagonists tested suggest that *in vitro* receptor occupancy may be longer lasting than would be predicted. This raises questions as to whether pharmacokinetic studies measuring plasma or brain levels of these antagonists are accurate reflections of receptor occupancy *in vivo*.

## Introduction

The orexin receptors, OX_1_R and OX_2_R, were deorphanised in 1998, when two independent teams identified the peptides orexin A and orexin B (de Lecea et al., 1998; Sakurai et al., 1998). OX_1_R and OX_2_R are G protein-coupled receptors that share 64% amino acid sequence identity in humans and are highly conserved between species (de Lecea et al., 1998; Sakurai et al., 1998). Both receptors can couple to G_q_ and mobilize intracellular Ca^2+^ via activation of phospholipase C (Sakurai et al., 1998), whilst OX_2_R can also couple G_i_/G_o_ and inhibit cAMP production via inhibition of adenylate cyclase (Zhu et al., 2003). In non-neuronal cells OX_2_R is capable of extracellular signal-regulated kinase activation via G_s_, G_q_, and G_i_ (Tang et al., 2008). In competition radioligand binding OX_1_R has a 10–100 fold higher affinity for orexin A (20 nM) than for orexin B (250 nM), whereas OX_2_R binds both orexin peptides with similar affinity (Sakurai et al., 1998).

Orexin is exclusively expressed by orexin producing neurons within the perifornical nucleus, the dorsomedial hypothalamic nucleus, and the dorsal and lateral hypothalamic areas (Peyron et al., 1998). Orexin producing neurons are limited to a few thousand in rodents, whereas in humans there are approximately 30,000–70,000. These neurons have both ascending and descending projections with dense projections to key nuclei of the ascending arousal system such as the adrenergic locus coeruleus, the serotonergic dorsal raphe, and the histaminergic tuberomammillary nucleus. These same regions also receive inhibitory projections from the ventrolateral preoptic area, which promote sleep (Sherin et al., 1998).

The orexin receptors are widely distributed in the brain in a pattern consistent with orexin neuron projections (Trivedi et al., 1998; Marcus et al., 2001). Although the expression patterns of the receptors are largely overlapping, OX_1_R is selectively expressed in the locus coeruleus and OX_2_R is expressed in the tuberomammillary nucleus. The broad distribution of the orexin system throughout the cortex, hippocampus, thalamic, and hypothalamic nuclei suggests it may modulate a variety of functions including arousal, appetite, metabolism, reward, stress, and autonomic function (Scammell and Winrow, 2011; Gotter et al., 2012).

Although orexin was originally named for its role in feeding behavior (Sakurai et al., 1998), the link between energy homeostasis and sleep/wakefulness is increasingly recognized (Yamanaka et al., 2003) and it is clear that the orexin system is crucial for the stability of wake and sleep states (Sakurai, 2007). The orexin system was first linked to the sleep disorder narcolepsy: a mutation in the OX_2_R gene was found to cause canine narcolepsy (Lin et al., 1999) and the knockout (KO) of orexin peptides in mice also resulted in narcolepsy with cataplexy (Chemelli et al., 1999). Indeed, several orexin system KO and transgenic models exhibit sleep abnormalities reminiscent of narcolepsy (Chemelli et al., 1999; Hara et al., 2001a,b; Willie et al., 2003; Beuckmann et al., 2004). The absence of orexin neurons or peptides and the double receptor KO mouse models recapitulate the human narcoleptic symptoms, with narcoleptic and cataplectic phenotypes, whereas single orexin receptor KO mice have only a moderate (OX_2_R) or no sleep phenotype (OX_1_R) (Chemelli et al., 1999; Scammell et al., 2000; Hara et al., 2001a,b; Beuckmann et al., 2002; Willie et al., 2003; Kalogiannis et al., 2011).

Narcolepsy with cataplexy is associated with severe daytime sleepiness (Tafti et al., 2005) due to the complete disorganization of the sleep/wake cycle, with sudden onset of Rapid Eye Movement (REM) sleep and cataplexy (loss of skeletal muscle tone without the loss of consciousness triggered by emotions). Patients with narcolepsy have undetectable levels of orexin in cerebral spinal fluid (Nishino et al., 2000) and a marked decrease in orexin producing cells in the hypothalamus (Thannickal et al., 2000). The cause of human narcolepsy is neurodegeneration of orexin-containing neurons, possibly due to an autoimmune disease (Tafti, 2007), although the precise mechanism is not established.

Not surprisingly, the orexin system has attracted substantial attention for the development of drugs for the treatment of insomnia. Dual orexin receptor antagonists or possibly selective OX_2_R antagonists are likely to be effective without some of the undesirable side effects of currently available treatments. Benzodiazepines and sedative hypnotics are commonly prescribed and inhibit arousal through activation or positive allosteric modulation of the GABA_A_ receptor. However, reported side effects include morning sedation, anxiety, anterograde amnesia, impaired balance and sleep behaviors such as sleep walking and eating (Buysse, 2013).

A number of orexin receptor antagonists have been developed that are expected to have advantages over classic sleep promoting drugs (see Uslaner et al., 2013). These have been reported as “dual” antagonists as they have apparently similar affinities for both OX_1_R and OX_2_R (Roecker and Coleman, 2008; Scammell and Winrow, 2011). Almorexant was the first compound for which clinical data was reported in volunteers and patients (Brisbare-Roch et al., 2007; Malherbe et al., 2009; Owen et al., 2009) followed closely by SB-649868 (also known as GW 649868) (Bettica et al., 2009a,b,2012a,b,c), suvorexant, the most advanced antagonist that has successfully completed phase III clinical trials (Cox et al., 2010; Winrow et al., 2011; Connor et al., 2012; Herring et al., 2012b; Ivgy-May et al., 2012) and filorexant (Coleman et al., 2012; Winrow et al., 2012). Also in this issue, we present our characterization of IPSU (Hoyer et al., 2013), an orally bioavailable, brain penetrant OX_2_R antagonist, on sleep architecture in mice.

**Figure 1:**
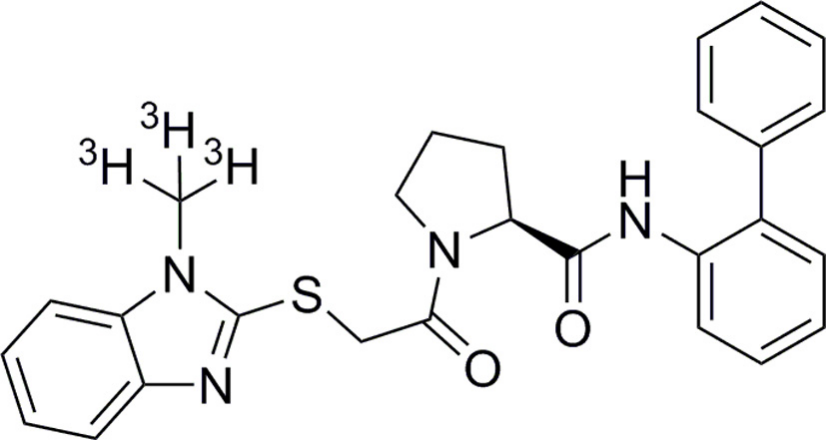
**Chemical structure of BBAC ((S)-N-([1,1′-biphenyl]-2-yl)-1-(2-((1-methyl-1H-benzo[d]imidazol-2-yl)thio)acetyl)pyrrolidine-2-carboxamide)**.

During the characterization of orexin receptor antagonists, we and others (Malherbe et al., 2010; Mang et al., 2012; Morairty et al., 2012) have noticed that almorexant has peculiar kinetic features, in particular a very slow dissociation rate constant especially at OX_2_R. Such features may be clinically relevant as they influence duration of action and potential for side effects. Therefore, we performed kinetic studies on the dual orexin receptor antagonists listed above in comparison with BBAC (Figure 1) and/or IPSU in radioligand binding and signaling studies at both OX_1_R and OX_2_R.

## Materials and methods

### Chemicals and reagents

[^3^H]-BBAC ((S)-N-([1,1′-biphenyl]-2-yl)-1-(2-((1-methyl-1H-benzo[d]imidazol-2-yl)thio)acetyl)pyrrolidine-2-carboxamide, Specific activity 73.76Ci/mmol) was synthesized at Novartis Pharma AG Basel (Isotope Laboratories). BBAC, SB-649868, suvorexant, filorexant, and IPSU (2-((1*H*-Indol-3-yl)methyl)-9-(4-methoxypyrimidin-2-yl)-2,9-diazaspiro[5.5]undecan-1-one) were synthesized at Novartis Pharma AG. Almorexant was synthesized by Anthem Biosciences (Bangalore, India).

### Cell culture and cell membrane preparation

Chinese Hamster Ovary (CHO) cells stably transfected with the cDNA encoding the human OX_1_R (CHO-hOX_1_) or OX_2_R (CHO-hOX_2_) were used (kindly provided by T. Cremer and Dr. S. Geisse, NIBR Basel, Switzerland). For measurements of calcium accumulation using FLIPR® (Fluorescent Imaging Plate Reader) assay, CHO or Human Embryonic Kidney (HEK) cells stably expressing mouse, rat or human OX_1_R or OX_2_R (kindly provided by Dr. A. Chen, GNF, San Diego, CA, USA) were used. All cells were cultured in 1:1 Dulbecco's Modified Eagle's Medium (DMEM)/Ham's F12 Nutrients Mixture (F12) supplemented with 10% (v/v) fetal bovine serum (FBS), 100 μ/ml (100 g/ml)/streptomycin (100 μg/ml), Fungizone (250 μg/ml), and Geneticin (G418, 50 mg/ml). Cells were maintained in a humidified incubator at 37°C in 5% CO_2_. For crude cell membrane preparations, cells were washed and harvested in 10 mM HEPES (pH 7.5), and centrifuged at 4°C for 5 min at 2500 g. The cell pellet was either stored at −80°C or used directly.

### Radioligand binding assays

Cell membranes were resuspended in binding assay buffer at 4°C (10 mM HEPES, pH 7.5, 0.5% (w/v) bovine serum albumin (BSA), 5 mM MgCl_2_, 1 mM CaCl_2_, and 0.05% Tween 20) and homogenized with a Polytron homogenizer at 50 Hz for 20 s. Cells were incubated with [^3^H]-BBAC in binding assay buffer in 96-deep well plates (Fisher Scientific). Aliquots of [^3^H]-BBAC were measured using liquid scintillation spectrometry on a LS 6500 scintillation counter (Beckman Coulter) to determine the amount radioactivity added to each well. Non-specific Binding (NSB) was determined in the presence of 1 μM almorexant. After the indicated incubation time, bound and free radioligand were separated by vacuum filtration using a Filtermate™ Cell Harvester (Perkin Elmer) and filtered onto 96-well deep GF/b filter plates (Millipore) which had been pre-treated with 0.5% (w/v) polyethyleneimine. Filter plates were rapidly washed three times with wash buffer (10 mM Tris-HCl, 154 mM NaCl, pH 7.4) at 4°C, dried and 25 μl of Microscint™ (Perkin Elmer) was added to each well. Radioactivity was quantified using a TopCount™ microplate counter (Perkin Elmer).

### Saturation binding

Binding was performed with eight concentrations of [^3^H]-BBAC (50 μl, 1–20 nM) to construct saturation curves. CHO-hOX_1_ or CHO-hOX_2_ cell membranes (150 μl/well) were incubated for 60 min in 96-deep well plates at room temperature with radioligand in binding assay buffer (50 μl) in the presence or absence of almorexant (1 μM, 50 μl), in a final volume of 250 μl. [^3^H]-BBAC binding was measured in triplicate in at least three independent experiments. Data in the figures is representative of the mean ± s.e.m. of a single experiment.

### Competition binding

Competition experiments were performed with a single concentration of radioligand and six concentrations of competitor (unlabeled ligands; BBAC, almorexant, SB-649868, suvorexant, filorexant or IPSU). 4.6 nM [^3^H]-BBAC (chosen from saturation experiments to provide 80–90% specific binding, 50 μl) was added simultaneously with various concentrations of unlabeled ligand (0.1 nM–10 μ M) to membranes (150 μl/well) in 50 μl/well of assay buffer with a total volume of 250 μl/well. The amount of [^3^H]-BBAC bound to receptors was determined at room temperature at different time points (ranging from 15 min to 4 h) and terminated by rapid vacuum filtration and liquid scintillation counting. Binding at a given concentration of competitor at a given time was measured in triplicate in at least three independent experiments. Data in figures is representative of the mean ± s.e.m. of a single experiment.

### Data analysis

All data was analyzed using GraphPad Prism 4.0 (GraphPad, San Diego, USA). The saturation data was fit to a non-linear regression model for saturation binding with consideration for one site binding. In addition, saturation binding data was also analyzed according to Scatchard (Scatchard, 1949; plots not shown). Competition binding data was fit to a non-linear regression model for competition binding with consideration for variable one site binding with a non-fixed Hill slope. The method of Cheng and Prusoff (1973) was used to convert IC_50_ values from competition binding curves to Ki (equilibrium dissociation constant) values.

### Functional analysis of dual antagonists on human orexin receptors

Determination of orexin A-stimulated calcium accumulation was performed over 2 days using FLIPR® (Fluorescent imaging plate reader from Molecular Devices-FLIPR384). Cells expressing either human, rat or mouse OX_1_R or OX_2_R were seeded at 8,000 cells/well in black 384 well clear bottom plates and incubated overnight at 37°C. The following day, medium was discarded and cells loaded with 50 μ l of 1 mM Fluo-4 AM (Invitrogen F14202) in dimethyl sulfoxide in working buffer (Hanks' balanced salt solution, 10 mM HEPES) and incubated for 60 min at 37°C. The loading buffer was removed and cells were washed with 100 μ l working buffer containing 200 mM CaCl_2_, 0.1% BSA, and 2.5 mM Probenecid (pH 7.4) to remove the excess Fluo-4 AM. Working buffer was added and plates were incubated 10–15 min at room temperature. The assay plate was then transferred to the Molecular Devices-FLIPR384. The baseline calcium signal was recorded for 10 s, then the antagonist of interest was injected (10 μl at 3 times the final concentration) and the calcium signal recorded every second for 1 min, then every 2 s 40 times. Plates were then incubated at room temperature for 30 min, 1, 2, or 4 h. Calcium signals were again measured as above, this time orexin A (15 μl) was injected at 3 times the final concentration. For each experiment, full orexin A concentration response curves were generated on each plate: they served to calculate the EC_50_ for that plate and to adapt the EC_80_ values in the subsequent experiments, which vary according to cell line and passage number.

The concentration response curves were analyzed according to the law of mass action, for both orexin A (EC_50_), and antagonists (IC_50_) with slope factors and maximal/minimal effects; the antagonist data was transformed according to Cheng and Prusoff (1973) (K_i_ = IC_50_/1 = (L/EC_50_)) where L is the agonist concentration used in the assay and EC_50_ its concentration for half maximal activation and the antagonist data was finally expressed as K_i_ (nM) and pK_i_ values (−log M).

## Results

### Time-dependent changes in apparent affinity as determined in radioligand binding

**Figure 2:**
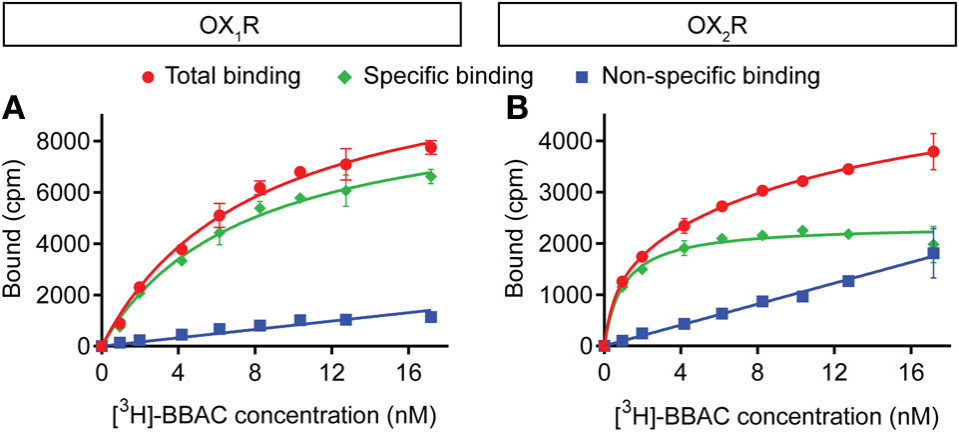
**Saturation binding of [^3^H]-BBAC ((S)-N-([1,1′-biphenyl]-2-yl)-1-(2-((1-methyl-1H-benzo[d]imidazol-2-yl)thio)acetyl)pyrrolidine-2-carboxamide) to membranes from CHO cells expressing human (A) OX_1_R or (B) OX_2_R**. Almorexant was used to define non-specific binding (blue). Total binding is indicated in red and specific binding in green. Data is representative of triplicate determinations and error bars indicate s.e.m.

[^3^H]-BBAC bound both OX_1_R and OX_2_R with high affinity and K_D_ values of about 7 nM and 1 nM, respectively (Figure 2). Binding reached equilibrium very quickly, as 15–30 min incubation time was sufficient to reach B_max_ and *K*_D_ values comparable to those measured after 4 h (data not shown).

**Figure 3:**
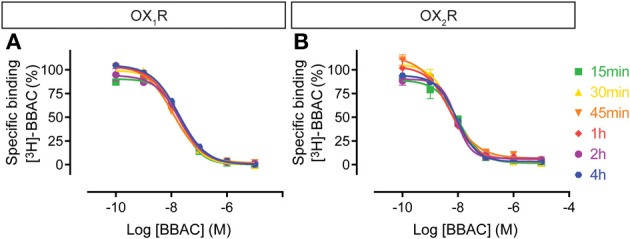
**Effect of time on BBAC ((S)-N-([1,1′-biphenyl]-2-yl)-1-(2-((1-methyl-1H-benzo[d]imidazol-2-yl)thio)acetyl)pyrrolidine-2-carboxamide) competition for [^3^H]-BBAC binding to membranes from CHO cells expressing human (A) OX_1_R or (B) OX_2_R**. Data is representative of triplicate determinations and error bars indicate s.e.m.

Competition experiments were performed with the various antagonists at 15, 30, 45 min, 1, 2, or 4 h and the graphs illustrate the competition curves at the different times. As expected from the saturation experiments described above, as well as in further kinetic experiments to be reported elsewhere, BBAC reached equilibrium quickly at both OX_1_R and OX_2_R (15–30 min), and there was no significant difference in IC_50_ values measured between 30 min and 4 h, as illustrated by superimposable competition curves at both orexin receptors (Figure 3).

**Figure 4:**
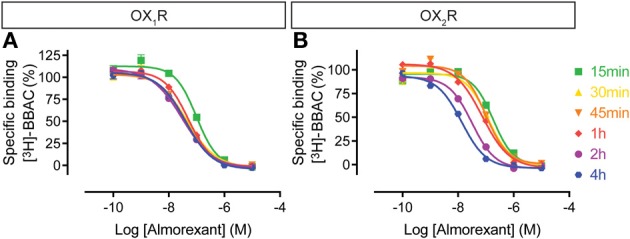
**Effect of time on almorexant competition for [^3^H]-BBAC ((S)-N-([1,1′-biphenyl]-2-yl)-1-(2-((1-methyl-1H-benzo[d]imidazol-2-yl)thio)acetyl)pyrrolidine-2-carboxamide) binding to membranes from CHO cells expressing human (A) OX_1_R or (B) OX_2_R**. Data is representative of triplicate determinations and error bars indicate s.e.m.

In contrast to BBAC, the competition curves for almorexant shifted to the left with time moderately at OX_1_R and substantially at OX_2_R (Figure 4). In other words, almorexant showed similar apparent affinity at OX_1_R between 30 min and 4 h, whereas the apparent affinity at OX_2_R increased up to 4 h of incubation. The data suggests that equilibrium at OX_2_R can only be reached after prolonged incubation, which also means that under short term conditions, almorexant is a dual orexin receptor antagonist, whereas after several hours of exposure, the compound becomes somewhat OX_2_R selective.

**Figure 5:**
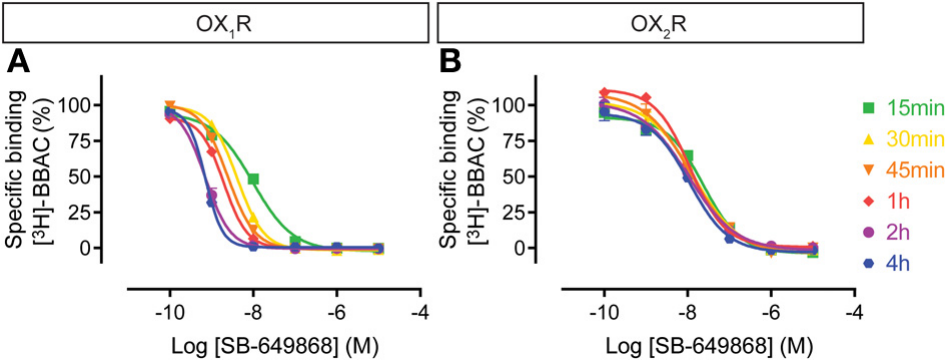
**Effect of time on SB-649868 competition for [^3^H]-BBAC ((S)-N-([1,1′-biphenyl]-2-yl)-1-(2-((1-methyl-1H-benzo[d]imidazol-2-yl)thio)acetyl)pyrrolidine-2-carboxamide) binding to membranes from CHO cells expressing human (A) OX_1_R or (B) OX_2_R**. Data is representative of triplicate determinations and error bars indicate s.e.m.

The SB-649868 competition curves on OX_1_R shifted to the left over time up to 4 h, whereas at OX_2_R binding appeared to be rather stable (Figure 5), suggesting that the compound equilibrated very rapidly at OX_2_R whereas it took hours to equilibrate at OX_1_R. This means that although acting as a dual antagonist acutely, given sufficient time to equilibrate, SB-649868 will show some OX_1_R selectivity.

**Figure 6:**
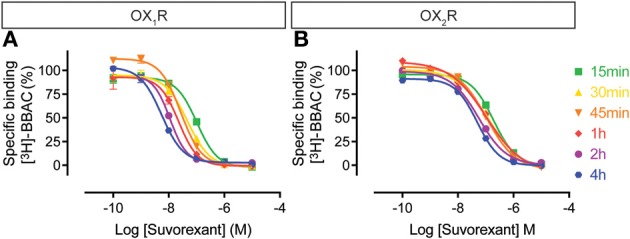
**Effect of time on suvorexant competition for [^3^H]-BBAC ((S)-N-([1,1′-biphenyl]-2-yl)-1-(2-((1-methyl-1H-benzo[d]imidazol-2-yl)thio)acetyl)pyrrolidine-2-carboxamide) binding to membranes from CHO cells expressing human (A) OX_1_R or (B) OX_2_R**. Data is representative of triplicate determinations and error bars indicate s.e.m.

Similarly, the suvorexant competition curves for both OX_1_R and OX_2_R shifted to the left over time, although the effect on OX_2_R was somewhat less pronounced (Figure 6). Thus, suvorexant equilibrates slowly at both orexin receptors and since equilibrium is generally driven by the dissociation rate constant, this means that once steady state binding is reached, receptor occupancy will be long lasting.

**Figure 7:**
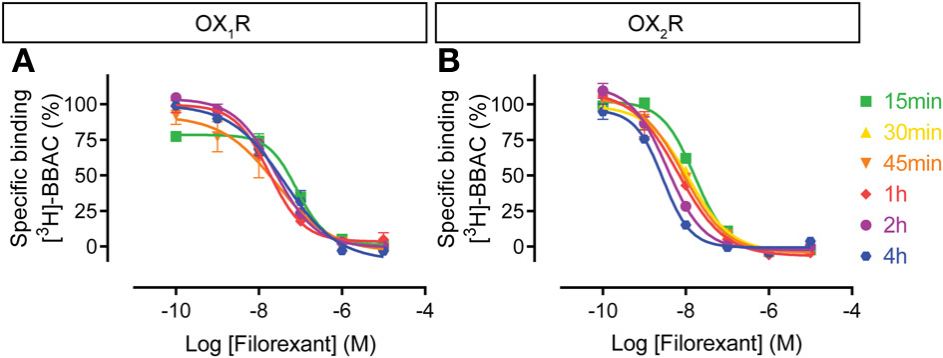
**Effect of time on filorexant competition for [^3^H]-BBAC ((S)-N-([1,1′-biphenyl]-2-yl)-1-(2-((1-methyl-1H-benzo[d]imidazol-2-yl)thio)acetyl)pyrrolidine-2-carboxamide) binding to membranes from CHO cells expressing human (A) OX_1_R or (B) OX_2_R**. Data is representative of triplicate determinations and error bars indicate s.e.m.

The filorexant competition curves at OX_1_R were rather insensitive to incubation time, whereas OX_2_R curves shifted to the left over time, even up to 4 h (Figure 7). Thus, similar to the other dual orexin receptor antagonists tested here, filorexant reaches equilibrium only after several hours of incubation, especially at OX_2_R.

**Figure 8:**
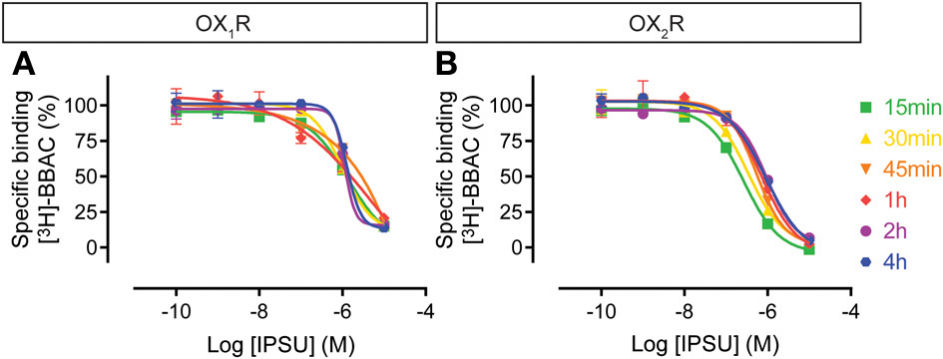
**Effect of time on IPSU competition for [^3^H]-BBAC ((S)-N-([1,1′-biphenyl]-2-yl)-1-(2-((1-methyl-1H-benzo[d]imidazol-2-yl)thio)acetyl)pyrrolidine-2-carboxamide) binding to membranes from CHO cells expressing human (A) OX_1_R or (B) OX_2_R**. Data is representative of triplicate determinations and error bars indicate s.e.m.

The IPSU competition curves at OX_1_R and OX_2_R do not show time-dependency (Figure 8), since maximal inhibition was already achieved following 15 min of incubation. This suggests a very rapid binding and equilibrium and a tendency to a rightward shift, suggesting faster kinetics than for the radioligand.

### Time-dependent changes in apparent affinity as determined in calcium assays

In the calcium accumulation assays performed at mouse, rat and human OX_1_R and OX_2_R, we first confirmed that orexin A produces stable results and that the apparent potency is largely comparable when the effects of antagonists are measured following incubation times of between 30 min and 4 h. Indeed, pEC_50_ values for orexin were largely time-independent at both OX_1_R and OX_2_R. This suggests the cells and receptors used were stable and would allow incubation times of up to 4 h in the subsequent experiments (Tables 1, 2). At OX_1_R, almorexant had an apparent antagonist potency which was constant, irrespective of the incubation time (30 min–4 h, Table 1). By contrast, at OX_2_R, the apparent potency kept increasing with incubation time (Table 2), as suggested by the radioligand binding experiments. These results indicate that across the three species studied here there is an apparent OX_2_R selectivity after longer incubation times. Filorexant showed time-independent potencies at OX_1_R, whereas at OX_2_R the apparent potencies increased with time. Suvorexant showed a time-dependent shift toward higher potency as time increased at both receptors, although the effects were more pronounced at OX_2_R. For SB-649868, antagonism at both receptors tended to increase with time, although the increase was greater at OX_1_R.

**Table 1:** **Ca^2+^ signaling in cells stably transfected with human (CHO), rat (HEK) or mouse (HEK) OX_1_R in the presence of the endogenous agonist (orexin A) or putative dual orexin receptor antagonists (almorexant, filorexant, suvorexant, SB-649868, and IPSU)**.

	**OX_1_R**											
	**30 min**			**1 h**			**2 h**			**4 h**		
	**pKi**	**s.e.m**.	***n***	**pKi**	**s.e.m**.	***n***	**pKi**	**s.e.m**.	***n***	**pKi**	**s.e.m**.	***n***
**HUMAN**												
Orexin A	9.52	0.10	3	9.48	0.01	541	9.51	0.07	3	9.63	0.10	3
Almorexant	7.84	0.05	6	7.73	0.06	26	7.75	0.07	6	7.75	0.07	6
Filorexant	8.65	0.09	6	8.79	0.08	12	8.78	0.11	6	8.55	0.06	6
Suvorexant	8.39	0.09	6	8.74	0.10	8	8.75	0.12	6	8.73	0.15	6
SB-649868	9.03	0.05	6	8.87	0.09	12	9.29	0.08	6	9.38	0.10	6
IPSU	6.32	0.08	6	6.29	0.05	6	6.28	0.09	6	6.14	0.08	6
**RAT**												
Orexin A	8.92	0.08	3	9.01	0.09	3	9.01	0.05	3	8.86	0.09	3
Almorexant	7.90	0.06	6	7.90	0.07	6	7.95	0.08	6	7.76	0.08	6
Filorexant	9.12	0.13	6	9.21	0.18	6	9.30	0.17	6	8.92	0.18	6
Suvorexant	8.82	0.20	6	9.40	0.36	6	9.37	0.19	6	9.24	0.17	6
SB-649868	9.59	0.11	6	9.90	0.19	6	10.17	0.10	6	10.14	0.06	6
IPSU	6.65	0.05	6	6.62	0.06	6	6.56	0.13	6	6.25	0.06	6
**MOUSE**												
Orexin A	9.17	0.14	3	9.18	0.04	31	9.36	0.15	3	9.25	0.14	3
Almorexant	7.73	0.05	6	7.63	0.10	10	7.47	0.07	6	7.41	0.11	4
Filorexant	8.28	0.10	6	8.39	0.14	9	8.18	0.08	6	8.23	0.08	6
Suvorexant	8.90	0.14	6	8.98	0.18	4	9.03	0.08	6	8.99	0.08	6
SB-649868	9.80	0.12	6	9.37	0.14	7	10.39	0.16	6	10.33	0.17	6
IPSU	6.48	0.04	6	6.31	0.12	4	6.54	0.06	6	6.49	0.06	6

**Table 2:** **Ca^2+^ signaling in cells stably transfected with human (CHO), rat (HEK) or mouse (HEK) OX_2_R in the presence of the endogenous agonist (orexin A) or putative dual orexin receptor antagonists (almorexant, filorexant, suvorexant, SB-649868, and IPSU)**.

	**OX_1_R**											
	**30 min**			**1 h**			**2 h**			**4 h**		
	**pKi**	**s.e.m**.	***n***	**pKi**	**s.e.m**.	***n***	**pKi**	**s.e.m**.	***n***	**pKi**	**s.e.m**.	***n***
**HUMAN**												
Orexin A	8.76	0.05	3	8.79	0.01	548	8.61	0.07	3	8.70	0.02	3
Almorexant	8.33	0.05	6	8.82	0.06	29	8.80	0.15	6	9.09	0.22	6
Filorexant	9.45	0.09	6	9.65	0.06	11	9.73	0.10	6	9.77	0.09	6
Suvorexant	9.00	0.14	6	9.48	0.14	8	9.46	0.19	6	9.53	0.20	6
SB-649868	9.52	0.05	6	9.43	0.09	15	9.77	0.03	6	9.82	0.05	6
IPSU	8.00	0.10	6	7.97	0.07	6	7.82	0.08	6	7.68	0.11	6
**RAT**												
Orexin A	8.40	0.01	3	8.34	0.07	6	8.48	0.06	3	8.58	0.03	3
Almorexant	8.25	0.08	6	8.65	0.06	6	8.99	0.19	6	9.18	0.10	6
Filorexant	9.13	0.11	6	9.38	0.08	6	9.60	0.11	6	9.75	0.10	6
Suvorexant	8.71	0.19	6	9.06	0.18	6	9.22	0.20	6	9.37	0.22	6
SB-649868	9.34	0.06	6	9.50	0.05	6	9.81	0.07	6	9.85	0.05	6
IPSU	7.63	0.14	6	7.55	0.11	6	7.62	0.12	6	7.55	0.11	6
**MOUSE**												
Orexin A	8.78	0.07	3	9.05	0.02	53	8.94	0.01	3	9.18	0.02	3
Almorexant	7.72	0.06	5	8.03	0.05	14	8.09	0.05	6	8.38	0.05	6
Filorexant	8.67	0.13	6	8.68	0.06	9	8.84	0.10	6	8.89	0.11	6
Suvorexant	7.99	0.11	6	8.17	0.14	4	8.24	0.11	6	8.35	0.08	6
SB-649868	8.74	0.07	6	8.55	0.08	7	8.93	0.06	6	9.04	0.04	6
IPSU	7.15	0.04	6	7.10	0.09	4	7.26	0.06	6	7.22	0.07	6

## Discussion

A thorough exploration of the pharmacokinetics and pharmacodynamics of drug candidates is important in drug development. Ideal sleep-enabling compounds have distinct profiles: rapid absorption and induction of sleep, low blood drug concentrations 8 h after dosing and efficacy in the absence of side effects (Wilson et al., 2010). Understanding the nuances of the kinetics of binding, such as the time taken to reach binding equilibrium, can provide valuable predictive information on duration of action and explain efficacy in patients.

With this in mind we sought to characterize the kinetic features of various “dual” orexin receptor antagonists at OX_1_R and OX_2_R. We selected antagonists that have either been used clinically or are currently under development for the treatment of insomnia and sleep disorders, including almorexant, SB-649868, suvorexant, and filorexant. We compared the kinetic features of these compounds with those of BBAC (a fast binding dual orexin receptor antagonist that was also used as a radioligand in the present studies) and IPSU, an OX_2_R antagonist (see Betschart et al., 2013; Hoyer et al., 2013). Our results show clearly that each of the ligands tested has different properties at both OX_1_R and OX_2_R, especially with respect to kinetics and suggest that at steady state each of these compounds has a pharmacological profile different from that measured under non-equilibrium conditions.

We observed that the radioligand [^3^H]-BBAC binds with high affinity, rapidly and reversibly to both OX_1_R and OX_2_R. In competition assays, unlabeled BBAC was a fast dual receptor binder, as illustrated by competition curves which are virtually superimposable irrespective of receptor type or incubation time. The slight shift to the right as time increased indicates the concentration dependence of the association rate, since the concentrations of unlabeled ligand used in the competition experiments (Figure 3; up to 10 μM) are higher than those used for the radioligand (low nM). This suggests unlabeled BBAC reaches apparent equilibrium faster than [^3^H]-BBAC.

For the dual orexin receptor antagonists tested, time-dependent changes in the apparent affinities for the receptors were found. The affinity of SB-649868 at hOX_1_R increased markedly between 15 min and 4 h, whilst time had little effect on the affinity at hOX_2_R (Figure 5). The opposite is true for almorexant, which displayed a leftward shift at hOX_1_R and a very pronounced increase in affinity at hOX_2_R as incubation time increased (Figure 4). Thus, SB-649868 and almorexant are slowly equilibrating antagonists, presumably because their dissociation rates are very slow. The data also suggests that when equilibrium is allowed to be reached, SB-649868 becomes somewhat hOX_1_R selective, whereas almorexant becomes hOX_2_R selective. The suvorexant competition curves demonstrated both hOX_1_R and hOX_2_R have increasing affinity with time, although the effect on hOX_2_R was somewhat less pronounced (Figure 6). Filorexant shows somewhat different properties, in that equilibrium was slow to be reached at hOX_2_R. By contrast, time had almost no effect on the affinity of both BBAC and IPSU as measured in the binding experiments. That is, the apparent affinity values measured at 15 min of incubation were at least as high as those measured after 4 h, an indication that they reach steady state at either receptor within a few minutes.

The time-dependent binding translated into differences in the more functional FLIPR® calcium assay in whole cells expressing human, rat, or mouse OX_1_ and OX_2_ receptors. Almorexant acted as a pseudo-irreversible or very slowly equilibrating antagonist at human, rat or mouse OX_2_R, whereas, at OX_1_R for all three species, almorexant behaved as a fast equilibrating antagonist. This data suggests that although originally described as a dual antagonist with very similar affinity for both receptors, almorexant is in fact a slowly equilibrating and somewhat selective OX_2_R antagonist, if sufficient time is given for the ligand to reach equilibrium. Similar findings were made in the calcium experiments with suvorexant, SB-649868 and filorexant, indicating that all display slow equilibration at one and/or the other orexin receptor (see Tables 1, 2). By contrast, IPSU (and BBAC) had constant potency values irrespective of the incubation time, again suggesting very fast equilibration at both orexin receptors. On the basis of both the radioligand binding and calcium accumulation data presented here, almorexant is likely to be OX_2_R selective, a finding that is in agreement with other reports that found almorexant to behave as a dual antagonist only during short incubation times (Malherbe et al., 2009; Mang et al., 2012; Morairty et al., 2012). In addition, we demonstrate in contrast to almorexant, SB-649868, suvorexant, and filorexant have a greater affinity for OX_1_R with long incubation times.

The differences in binding kinetics between the orexin receptor antagonists demonstrated here are likely to have implications for pharmacodynamics. Suvorexant is a pertinent example: studies of pharmacokinetics revealed a long dose-dependent apparent terminal half-life (between 9 and 12 h, Merck Sharp and Dohme Corporation, 2013a) and next morning residual effects (Sun et al., 2013). It is possible that these residual effects are not only related to half-life, but also longer than expected target/exposure engagement. In addition, the suggestion that suvorexant has a tendency to accumulate after 4 weeks of consecutive treatment is not surprising (Farkas, 2013) given 24 h following administration of a single dose, mean plasma levels of suvorexant remain between 0.1 and 0.6 μ M (Sun et al., 2013). These results may be explained by a combination of pharmacokinetic effects (slow elimination or metabolism) and pharmacodynamic effects (slow equilibration and off rates), as shown in the present studies.

The Food and Drug Administration (FDA, USA) have concluded that although suvorexant is efficacious, it is not considered safe at doses higher than 20 mg (Farkas, 2013). The key safety concerns raised were rapid onset daytime somnolence, motor impairment, driving impairment, unconscious night time activity such as sleep walking, suicidal ideation, hypnogogic hallucinations and effects resembling mild cataplexy (Farkas, 2013; Radl, 2013; Sun et al., 2013). All of these appeared to be dose and plasma-exposure dependent. In addition, the FDA suggested an effort to find the lowest effective dose may be warranted (Farkas, 2013). Merck has determined that additional clinical studies are not necessary for the 10 mg dose, however, may be required to support a 5 mg dose (Farkas, 2013; Merck Sharp and Dohme Corporation, 2013b).

The individual contribution of orexin receptors to sleep architecture is a matter of debate since, to our knowledge, no selective OX_1_R or OX_2_R antagonist has been tested in patients with insomnia. However, rodent models are rather good predictors of the effects of orexin receptor antagonists on sleep. In rodents, OX_2_R antagonism appears sufficient to induce sleep: almorexant is effective in the OX_1_R KO whereas it has no effect on the sleep wake cycle in OX_2_R or in double receptor KO mice (Mang et al., 2012). Further, in rodents with targeted destruction of the orexin neurons of the lateral hypothalamus, treatment with almorexant tends to induce cataplexy (Black et al., 2013). Nevertheless, there are major differences relating to pharmacokinetics and pharmacodynamics between species. One should also keep in mind that whilst narcoleptic/cataplectic dogs have a defect in OX_2_R, this has never been observed in humans.

In addition to almorexant, SB-649868 and suvorexant have reached phase II clinical trials for the treatment of insomnia. Clinical data suggests that the main effect on total sleep time is largely due to an increase in REM sleep and decreased latency to REM, with modest effects on non-REM or slow wave sleep, if at all (Bettica et al., 2012a,c; Herring et al., 2012a,b; Hoever et al., 2012). In the case of SB-649868, there is strong evidence of sleep onset REM in patients receiving the 60 mg dose (Bettica et al., 2012a). Whilst no clinical evidence exists for filorexant, recent rodent studies demonstrated the filorexant analog, DORA-22, promotes sleep with dose-dependent increases in REM sleep (Fox et al., 2013), suggesting that the mechanism may also be the same for this compound.

Overall, the clinical data appears to confirm the preclinical data collected in mice or rats which demonstrates dual orexin receptor antagonists or dual receptor KOs induce sleep with a very strong REM component, whereas OX_2_R KO or antagonism has more balanced sleep phenotypes (Willie et al., 2003; Mang et al., 2012; Betschart et al., 2013; Hoyer et al., 2013). Therefore, one may consider OX_1_R antagonism to be detrimental and suggest that compounds such as suvorexant and SB-649868, which show very slow kinetics at the OX_1_R, are likely to favor REM over non-REM. For SB-649868, clinical studies in healthy volunteers (Bettica et al., 2012b) and insomnia patients (Bettica et al., 2012c) demonstrate that 10, 30, or 60 mg SB-649868 decreases latency to REM and increases REM duration. In a 4 week placebo-controlled study of suvorexant in patients with insomnia, Herring and colleagues observed that increases in total sleep time were mainly due to increased time spent in REM sleep (Herring et al., 2012b). Such compounds may also increase rapid transitions between wake and REM states, especially if the compound is given a relatively long time before bed, as was the case with SB-649868 (90 min, Bettica et al., 2009a,2012b).

Still, kinetics are of primary importance in sleep and an appropriate balance must be reached for therapeutic efficacy and safety. If target occupancy is too short, the patient will wake up in the middle of the night as happened with early formulations of Z drugs such as zolpidem and zaleplon (Besset et al., 1995; Roth et al., 1995; Greenblatt et al., 1998). Conversely, if target occupancy is too long, there will be “hangover” effects into the next morning, a crucial issue with benzodiazepine hypnotics (Wilson et al., 2010). For compounds that have slow receptor kinetics, pharmacodynamics and pharmacokinetics may not run in parallel, complicating their further development. The current report suggests that all four established “dual” antagonists have very slow kinetics, leading to changes in actual selectivity if equilibrium can be reached *in vivo*; in addition, if equilibrium is reached, slow off rates may result in longer receptor occupancy than may be predicted solely from the pharmacokinetic data.

## Author contributions

Gabrielle E. Callander prepared the data for publication and wrote the manuscript. Morenike Olorunda, Dominique Monna, Edi Schuepbach, and Daniel Langenegger have carried out the experiments, contributed to the development of the assays and performed some of the data analysis. Claudia Betschart and Samuel Hintermann have synthesized a number of compounds and led the chemistry efforts and contributed to writing. Emmanuelle Briard contributed to the synthesis of a number of radioligands used in these studies. Dirk Behnke, Simona Cotesta, Grit Laue, and Silvio Ofner have synthesized other compounds and/or have performed a number of analyses in relation to pharmacokinetics. Christine E. Gee, Markus Fendt, and Laura H. Jacobson have performed *in vivo* experiments that led to the concepts developed here and have participated in discussion and writing. Daniel Hoyer has led the team, conceptualized the experimental approach and data interpretation and finalized the writing.

### Conflict of interest statement

With the exception of Gabrielle E. Callander, the authors are either past or present Novartis employees, or have been supported by Novartis.

## Abbreviations

5-HT: serotonin
BBAC: (S)-N-([1,1′-biphenyl]-2-yl)-1-(2-((1-methyl-1H-benzo[d]imidazol-2-yl)thio)acetyl)pyrrolidine-2-carboxamide
BSA: Bovine Serum Albumin
cAMP: cyclic AMP
CHO: Chinese Hamster Ovary
DMEM: Dulbecco's Modified Eagle's Medium
F12: Ham's F12 nutrients mixture
FDA: Federal Drug Administration of the United States Department of Health and Human Services
FLIPR®: FLuorescent Imaging Plate Reader
GABA: γ-aminobutyric acid
HEK: Human Embryonic Kidney
IPSU: 2-((1*H*-Indol-3-yl)methyl)-9-(4-methoxypyrimidin-2-yl)-2,9-diazaspiro[5.5]undecan-1-one
KO: Knock Out
NSB: Non-specific Binding
OX_1_R: orexin receptor 1
OX_2_R: orexin receptor 2
REM: Rapid Eye Movement (sleep state).
